# “Crocus Flower”: Voriconazole-Induced Hallucinations and Visual Disturbances in a Patient with Recurrent Severe Vulvovaginitis—A Case Report on Irrational Drug Use

**DOI:** 10.3390/reports7040105

**Published:** 2024-11-22

**Authors:** Svetoslav Stoev, Hristina Lebanova

**Affiliations:** Department of Pharmaceutical Sciences and Social Pharmacy, Faculty of Pharmacy, Medical University Pleven, 5800 Pleven, Bulgaria

**Keywords:** voriconazole, visual disturbances, infusion rate, adverse drug reactions, clinical pharmacy

## Abstract

**Background and Clinical Significance:** Voriconazole is a commonly prescribed second-generation azole used for the prevention and treatment of fungal infections. This report seeks to elucidate the relationship between certain intravenous infusion parameters and the causality and severity of potential visual adverse events associated with voriconazole administration, despite existing reports of visual disturbances such as hallucinations and altered visual perception, the underlying causes of which remain inadequately understood. **Case Presentation:** This case report describes a 32-year-old female patient who experienced sudden hallucinations and visual impairments after receiving an initial dose of intravenous voriconazole for the treatment of recurrent severe vulvovaginitis caused by *Candida glabrata*. The symptoms quickly disappeared when the dosage and infusion rate were reduced as per the recommendations of the clinical pharmacist. **Conclusions:** This example emphasizes the possible negative drug responses linked to voriconazole, especially those provoked by its irrational use described as an inappropriate infusion rate, and the crucial role of clinical pharmacists in recognizing and handling these reactions.

## 1. Introduction and Clinical Significance

Voriconazole is a routinely prescribed second-generation azole that is widely utilized for the prevention and treatment of fungal infections. There have been limited instances of adult patients reporting hallucinations and visual anomalies due to the administration of voriconazole. While there have been several reports detailing visual disturbances such as hallucinations, sensitivity to light, and changes in visual perception caused by alterations in the electroretinogram (ERG) or toxic optic neuropathy, the underlying causes of these occurrences are not yet fully understood [1]. This case report presents the clinical details of a 32-year-old female patient who exhibited symptoms following the intravenous administration of voriconazole. The symptoms were effectively alleviated by modifying the dosage and infusion rate as a result of the clinical pharmacist’s intervention. The pharmacist investigated the circumstances associated with the disease and identified the underlying cause of the stated visual impairment.

Through sharing this case report, our goal is to emphasize the proactive role that pharmacists may have in ensuring drug safety and optimizing therapy, ultimately leading to the progress of hospital pharmacy practice. 

The aforementioned case scenario does not include the involvement of the patient in a clinical trial or any other research activity. The subject underwent therapy following the established standard clinical practice and the enforced treatment guidelines, as recommended by the treating physician and under the responsibility of the treatment team.

## 2. Case Presentation

### 2.1. Patient Medical History and Case Background

A 32-year-old female (body mass index 20) was admitted to a specialized obstetrics and gynecology hospital in Sofia, Bulgaria, with a history of recurrent severe vulvovaginitis microbiologically confirmed to be caused by *Candida glabrata*. Prior therapeutic interventions, including both systemic and local approaches, had proven futile. However, the patient had previously been hospitalized for pharmaceutical treatment of the candidiasis infection. At the time of admission, she had been prescribed intravenous voriconazole (Vfend^®^, Pfizer, Purrs, Belgium) at 6 mg per kilogram every 12 h on the first day and 4 mg/kg once daily for the following four days. In addition, the advice from the treating physician involved taking oral fluconazole (Mycomax^®^, Zentiva, Prague, Czech Republic) at a dosage of 150 mg once daily for a duration of ten days after the voriconazole infusion. Additionally, butoconazole 20 mg/g (Gynazole^®^ vaginal cream, Gedeon Richter, Budapest, Hungary) had been prescribed to be used vaginally once every thirty days for a duration of 3 months following voriconazole treatment. The adjuvant therapy was supported by an oral probiotic product containing *Bacillus coagulans* microorganisms at a dose of one capsule daily for 6 months. 

### 2.2. Case Description

During the first day of voriconazole treatment, the subject experienced acute hallucinations and visual disturbances while receiving the infusion. The patient reported seeing a large area filled with crocus flowers and having vision that appeared in hues of purple. The female subject abruptly expressed her observation of a “field abundant with crocus flowers” seen through the hospital room window. She was taken aback when she observed that everything was tinted in various shades of purple. Due to the treating physician being overwhelmed and occupied at that specific moment, the clinical pharmacist took the initiative to attend to the patient and gather anamnestic information regarding their unusual state. However, the individual exhibited consciousness and full orientation in the correct moment, indicating her awareness. 

No further medications, including substances that may have disrupted the normal functioning of the central nervous system and caused visual disturbances, or chronic illnesses were reported. The patient had no prior psychiatric history and had never reported experiencing similar symptoms before. Following the clinical pharmacist’s recommendation, the voriconazole dose was decreased to 4 mg/kg given once daily, resulting in the total resolution of hallucinations and visual abnormalities. The voriconazole regimen was improved by decreasing the infusion rate from 3 mg/kg per hour to 2 mg/kg per hour while maintaining the same therapeutic results.

### 2.3. Investigations

**Figure 1:**
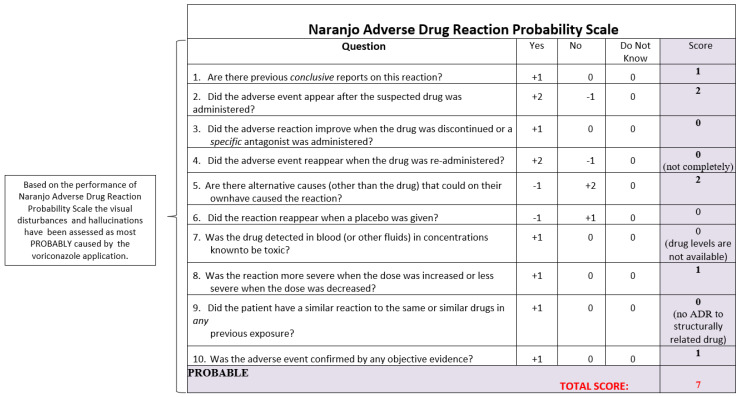
Probability assessment of the observed ADR, modified from Naranjo CA et al. A method for estimating the probability of adverse drug reactions. *Clin. Pharmacol. Ther.* **1981**, *30*, 239245. Reprinted with permission from Ref. [2]. Copyright 1981 Hoboken, NJ, USA John Wiley and Sons, License Number 5893661118754.

The clinical pharmacist gathered comprehensive anamnestic information through a detailed patient interview. A comprehensive assessment of potential drug interactions, including the prescription history from the past few weeks, was conducted. This assessment specifically looked for drug–drug interactions, drug–food interactions, and any delayed side effects of medications other than voriconazole. The Naranjo algorithm was used to evaluate the causal relationship between the observed hallucinations of crocus blossoms as well as the visual disturbances of a purple color, and the infusion of voriconazole (Figure 1). A blood chemistry and biochemical examination was conducted, but no clinically important findings were identified. The levels of Alanine Aminotransferase (ALAT) and Aspartate Aminotransferase (ASAT) showed a modest increase, but remained within the upper limit of the normal range. The patient underwent an evaluation for electrolyte imbalances, such as hypokalemia, hypomagnesemia, and hypocalcemia, but no issues of clinical significance were detected.

### 2.4. Treatment

The voriconazole regimen was enhanced by decreasing the rate of infusion from 3 mg/kg per hour to 2 mg/kg per hour while maintaining the effectiveness of the treatment. The person did not reveal any further occurrences of hallucinations, and the slow fading of the purple tones in their vision was documented. The clinical pharmacist diligently monitored whether both the nursing staff and the subject herself observed the reduced infusion rate. The nurse documented that the patient tried to expedite the administration of the medication to be released from the hospital sooner by applying pressure to the medication bag the day before, which is when the hallucinations took place.

### 2.5. Outcome and Follow-Up

The symptoms have shown improvement when the therapy has been maintained at a reduced dosage and corrected infusion rate, guaranteeing a slower administration of voriconazole and ensuring voriconazole blood levels remain within a safe range. Therapeutic drug monitoring (TDM) has not been utilized in this case due to the hospital’s inability to conduct it, and TDM is not a widely adopted technique in Bulgaria. The treating team chose to continue the voriconazole infusion, despite the Naranjo algorithm suggesting a possible link between the use of voriconazole and negative effects on eyesight [2]. Based on a consultation with the clinical pharmacist, it was determined that the adverse reaction was due to the unexpected intolerance of standard infusion parameters for voriconazole application, and the probable variation in the patient’s *CYP2C19* metabolizing capabilities should be taken into account for future treatments. A patient-tailored rationalization of parameters of intravenous administration of voriconazole led to the remission of symptoms.

## 3. Discussion

While hallucinations have been included in the summary of product characteristics (SmPC) as a possible adverse effect of voriconazole treatment, and several reports documenting visual impairment have been published, there is no documented instance in the literature of a patient reporting a specific visual adverse consequence such as seeing a field of crocus blossoms. Given that visual impairments and hallucinations tend to occur when voriconazole is administered, it is crucial to thoroughly evaluate the underlying cause of these visual side effects. Using the Naranjo Adverse Drug Reaction Probability Scale, it may be determined that the visual disturbances and hallucinations in this case were most likely caused by the use of voriconazole (Figure 1) [2]. 

A point worth considering is the voriconazole pharmacokinetic profile and pharmacodynamic pathways. Voriconazole is metabolized by *CYP2C19*, *CYP2C9*, and *CYP3A4* [1]. The *CYP2C19* gene causes voriconazole-treated individuals to have vastly different medication serum concentrations, so some instances of voriconazole-related hallucinations can be attributed to drug–drug interactions that impair the enzyme’s ability to metabolize voriconazole effectively [3]. *CYP2C19* poor metabolizers have 4-fold greater voriconazole exposure than normal metabolizers [4]. The Vfend^®^ summary of product characteristics acknowledges *CYP2C19*’s effect on drug levels but does not provide dose recommendations depending on metabolizer status. Only infection type and weight are included in the dosing instructions. However, some professional societies like the Dutch Pharmacogenetics Working Group (DPWG) and the Clinical Pharmacogenetics Implementation Consortium (CPIC) suggest voriconazole dosing should be based on the *CYP2C19* metabolizer type [4,5]. Both advisory documents suggest using an alternative agent that is not reliant on *CYP2C19* metabolism as the main treatment instead of voriconazole. However, if voriconazole is deemed the most suitable option based on clinical advice for an individual with a poor metabolizer genotype, it should be administered at a lower-than-standard dosage with careful monitoring of the drug’s effectiveness. In our case, genotype testing was not conducted either prior to commencing the treatment or following the sudden occurrence of crocus flower hallucinations and the purple visual impairments. 

A study examining the correlation between voriconazole-induced visual hallucinations and dopamine levels, using data from the Food and Drug Administration Adverse Event Reporting System (FAERS), concluded that there is a potential link between dopaminergic medication and an increased risk of visual hallucinations in patients undergoing voriconazole treatment [6]. However, we have received no reports of concurrent use of dopaminergic medication (levodopa) or dopamine antagonists (risperidone and chlorpromazine), nor have any documented nervous system diseases been described as potential prerequisites for visual impairment and hallucinations. 

The instance mentioned above bears resemblance to a series of cases documented in a study conducted by Zonios et al., in which 12 out of 72 patients treated with voriconazole developed hallucinations while undergoing voriconazole therapy. However, our case differs slightly as our subject observed the crocus field while fully conscious and awake, with her eyes open. She experienced the sensation of a genuinely beautiful view out of the hospital window, rather than hallucinating with closed eyes or during sleep, as observed in most cases in the referenced study. In our situation, the symptoms were quickly cured after the initial dose, and most crucially, after adjusting the infusion rate and dosage [7].

Our case is distinct from previously documented instances of visual impairments that have been reported after voriconazole infusion, which typically occur in children, underweight female individuals, immunocompromised people or patients with malignancies [8,9,10,11,12]. The adult woman in our case had a normal body mass index (BMI) and had not reported any chronic or metabolic conditions. It can be inferred that visual abnormalities and hallucinations caused by voriconazole have been documented when either taken orally or intravenously, with the latter being more frequently observed after intravenous infusion. The majority of published reports corroborate our hypothesis that hallucinations and visual anomalies are associated with the drug’s concentration in the plasma and/or cerebrospinal fluid. However, we hypothesize that the crucial aspect is precisely controlling the infusion parameters to prevent the occurrence of toxic and hallucination-related peaks, regardless of whether the correct dosage of voriconazole is delivered. A more individualized strategy should be applied to each case, as evidenced by the published research, which identifies many predisposing factors that contribute to the nerve toxicity of voriconazole and visual adverse consequences. 

Nonetheless, a limitation of the current reported information to consider is the potential gender-specific alteration of the pharmacokinetic properties of voriconazole. Despite contentious data concerning the direct correlation between gender and voriconazole metabolism, most published research findings, particularly those derived from extensive registry data, support the evidence of female-specific variations in voriconazole bioavailability [9,12,13].

Regrettably, due to the aforementioned circumstances that therapeutic drug monitoring (TDM) has not been implemented for the current subjects owing to insufficient hospital capacity and the minimal integration of bioavailability assessment as a standard practice in Bulgaria, it is not feasible to accurately correlate the incidence of adverse effects with the precise plasma levels of voriconazole to chronologically delineate the toxic pathway for the specific patient. However, the present case report corroborates the previously advised clinical protocols for rationalizing voriconazole utilization. The current findings support the recommendation that therapeutic drug monitoring and pre-emptive pharmacogenetic testing for *CYP2C19* are advisable to ensure that an appropriate voriconazole regimen is described [4,12,14] and to facilitate any necessary dose adjustment in both specific populations, such as children and hematology patients, and the general adult population. Furthermore, genotyping for anomalies in the *ABCC2* gene, which may affect the metabolism of voriconazole via multi-drug resistance proteins, could substantially alter clinical practice in the future. Therapeutic drug monitoring (TDM) has not been utilized in this case due to the hospital’s inability to conduct it, and because TDM is not a widely adopted technique in Bulgaria.

This occurrence of acute voriconazole-induced hallucinations and visual abnormalities demonstrates the potential seriousness and unpredictability of adverse drug reactions (ADRs) associated with voriconazole. When using voriconazole, clinical/hospital pharmacists and other healthcare professionals should carefully consider several factors, including the method of administration, rate of intravenous infusion, duration of infusion, and the patient’s medication history. It is crucial to take into account these infusion parameters, particularly in countries where therapeutic drug monitoring is not commonly implemented as a standard practice of care. In complex cases such as severe recurring vulvovaginitis, the appropriate administration of voriconazole is one of the few available ways to resolve the patient’s condition. Hospital pharmacists play a crucial role in promptly identifying these symptoms and taking appropriate corrective measures, such as adjusting medication dosages and infusion rates. Clinical pharmacy interventions can not only help resolve symptoms and improve patient outcomes without stopping antifungal therapy, but they can also be a valuable tool for optimizing patient treatment and preventing adverse events from happening in the first place. This method not only maintained the therapeutic effectiveness of voriconazole but also averted additional difficulties and discomfort for the patient who encountered the described condition. 

Moreover, sharing information on current voriconazole-related experiences will help increase our understanding of the drug’s characteristics and the unusual adverse effects it may cause. This compass would provide valuable guidance for hospital pharmacists in selecting the appropriate patient population for voriconazole treatment and identifying the individuals in whom and scenarios in which prescribing voriconazole medication according to the usual regimen without adjusting the dose or infusion rate is not recommended. This paper provides healthcare professionals, specifically pharmacists, with information regarding the possible adverse drug reactions (ADRs) linked to voriconazole and emphasizes the significance of vigilant monitoring throughout its administration. This demonstrates a concrete instance of how clinical pharmacists may proficiently handle adverse drug reactions (ADRs) by optimizing medication dosage and providing counseling to patients. As a result, patient care and safety are enhanced. This statement emphasizes the importance of the clinical pharmacist’s position in a healthcare team that consists of professionals from several disciplines. It demonstrates how the pharmacist’s specialized knowledge and skills can result in improved outcomes for patients. Ultimately, this report contributes to the current pool of information regarding adverse drug reactions (ADRs) caused by voriconazole. It offers valuable insights and viable approaches for handling similar cases in the future.

## 4. Conclusions

Clinical pharmacy contribution: Clinical pharmacists are crucial in promptly identifying and managing adverse drug responses to medications with unique pharmacokinetic profiles and narrow therapeutic windows, such as voriconazole. Clinical pharmacists’ interventions are an effective means of guaranteeing prompt and appropriate actions to alleviate patient suffering and prevent complications arising from unusual adverse outcomes of voriconazole infusion. 

Dose optimization: Modifying the dosage and rate of medication administration, as exemplified in this scenario, can successfully address peculiar adverse drug reactions (ADRs) associated with voriconazole without requiring cessation of treatment. This underscores the significance of personalized medicine in the practice of hospital pharmacy.

Monitoring of voriconazole treatment: Continuous monitoring and assessment of patients using high-risk drugs such as voriconazole are essential for rapidly discovering and resolving potential side effects, reaffirming the pharmacist’s responsibility in ensuring patient safety. 

Inpatient therapy: The results above show that it is advisable to initiate voriconazole treatment in hospital settings and patients receiving voriconazole infusion should be instructed not to drive following administration of the medication.

## Data Availability

The original contributions presented in this study are included in the article. Further inquiries can be directed to the corresponding author.
